# The Practical Medicine Series for 1910

**Published:** 1910-12

**Authors:** 


### Books and Magazines.

---

THE PRACTICAL MEDICINE SERIES FOR 1910.—Comprising ten volumes (seven now ready) on the year's progress in medicine and surgery. Under the general editorial charge of Gustavus P. Head, M. D., Professor of Laryngology and Rhinology, Chicago Post-Graduate Medical School; Charles L. Mix, A. M., M. D., Professor of Physical Diagnosis in the Northwestern University Medical School; assisted by Isaac A. Abt, M. D., Clinical Professor of Pediatrics, Northwestern University Medical School, with the collaboration of May Michael, M. D., John Ridlon, A. M., M. D., Professor of Orthopedic Surgery, Rush Medical College, with the collaboration of Charles A. Parker, M. D., also by Dudley, Bachelle, Billings, Murphy, De Lee, Wood, Andrews, Salisbury. Chicago: The Year-Book Publishers, 40 Dearborn Street. Cloth and gilt, $10 for the set of ten. Any volume sold separately. See price given below.

Volumes I to VII now ready. The remaining three to complete the set will be ready for delivery by January 1st—the ten volumes in twelve months. This work is published every year, primarily for the general practitioner, at the same time the arrangement in several volumes—classified, enables those interested in any special subject to buy only the volume or volumes they desire. Of course, separate volumes cost a little more than the average per volume, where the entire series is ordered; for instance, the volume on Surgery by the great Jno. B. Murphy, if sold separately, is $2 (Vol. II). Those on General Medicine, Billings & Salisbury (Vols. I and VI), $1.50 each. Eye, Ear, Nose and Throat by Wood, Andrews and Head, $1.50 each (Vol. III). Obstetrics by De Lee (Vol. V), $1.25. Gynecology, Dudley, Bachelle (Vol. IV), $1.25. Pediatrics, Abt, Ridlow (Vol. VII), $1.25 each, and so forth.

This series occupies a unique place in medical literature. It is mid-way between the monthly or weekly medical journals and the text-books or large monographs, which only appear a year or years after the subject matter on any special subject has been given the profession in the journals. It is an arrangement greatly appreciated and, in consequence, the work has an immense sale. Every doctor who loves his profession and therefore desires to keep step to the music of progress in medicine should subscribe for it. It is a work involving almost incredible research labor, as the editors must go through hundreds of medical journals every month, covering the whole domain of medicine, and cull out, abstract, edit and publish and disseminate everything new or important, as fast as it appears, thus enabling the doctor to have it in compact and permanent form in his library ready for reference—to any subject—in a moment. A double crossed index in each volume facilitates this. Besides, the set is quite ornamental and shows up to great advantage in the office. It is a monumental work every year, and the able and distinguished and painstaking editors deserve great credit for it, as do also the publishers, for, mechanically, the work is a *peach*, or, rather, a basket of them.—D.

